# Supplementary figures and images for: Characteristics and impact of Long Covid: Findings from an online survey
Source: PLoS One. 2022 Mar 8;17(3):e0264331. doi: 10.1371/journal.pone.0264331 (PMC8903286; doi:10.1371/journal.pone.0264331)

**S1 Fig: Reported SARS-CoV-2 testing history in survey participants**

**
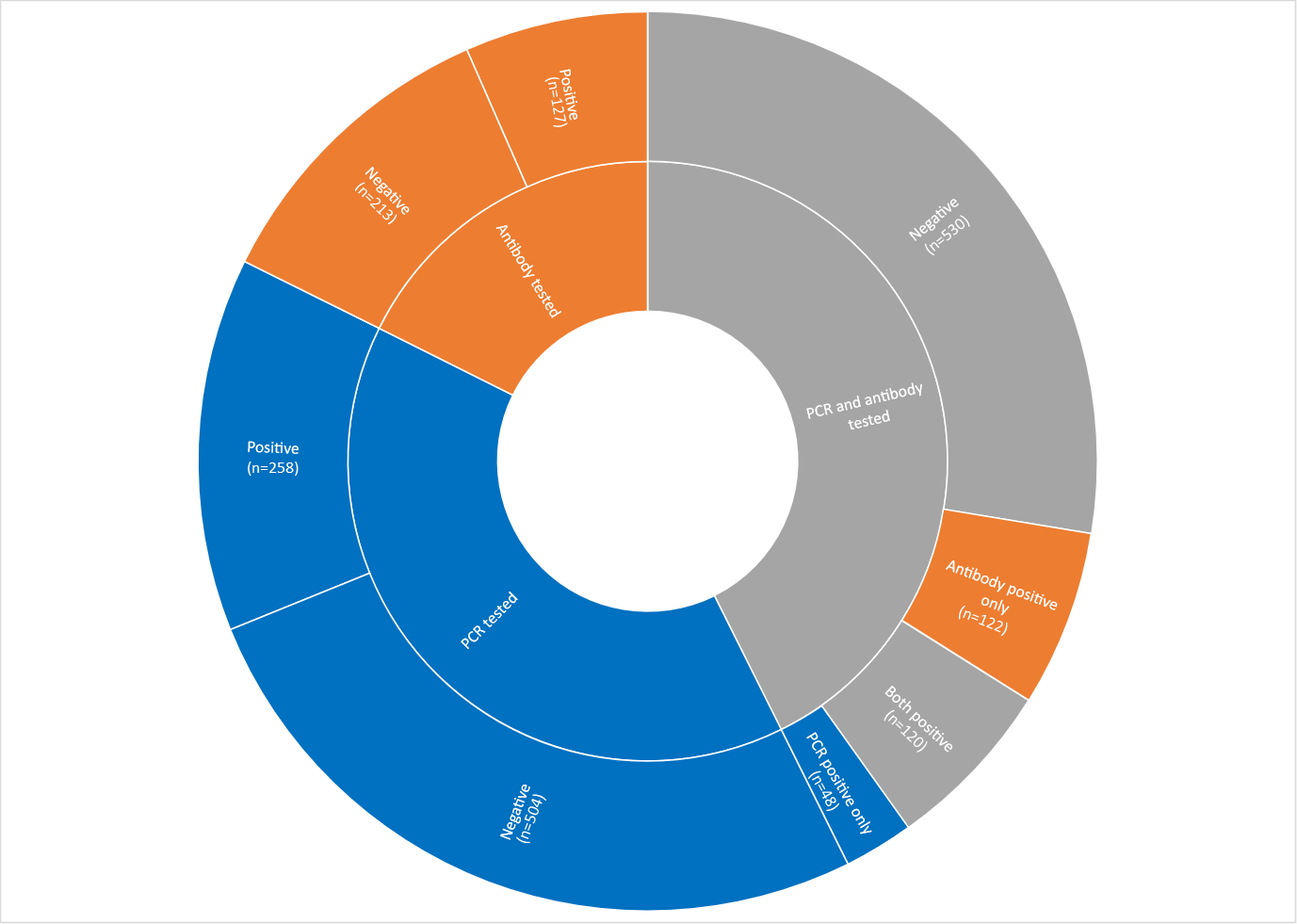
**

Supplement: S1 Fig — (DOCX) [file pone.0264331.s001.docx]

**S2 Fig: Silhouette coefficient for 2 to 10 clusters**


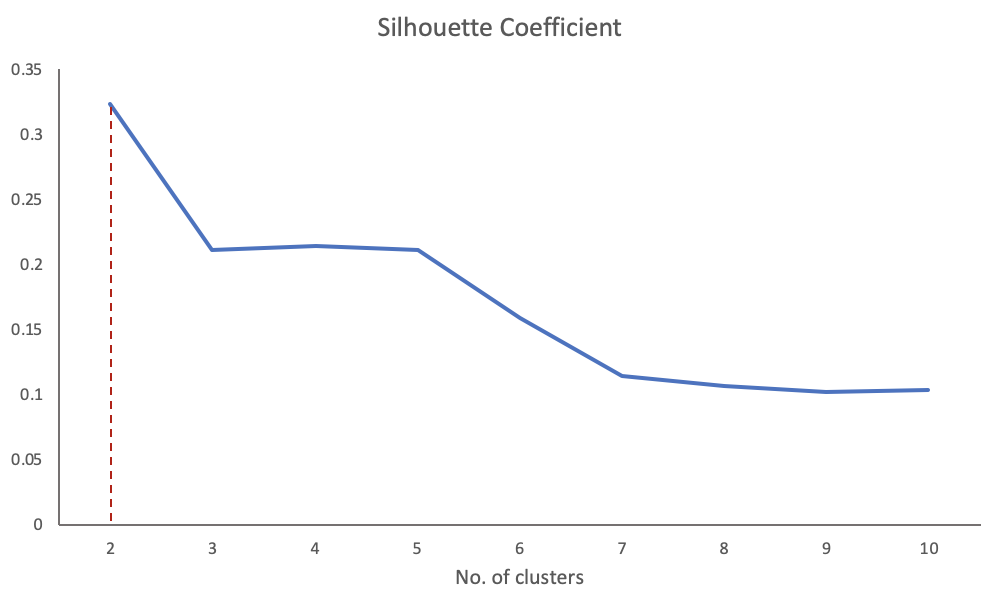

Supplement: S2 Fig — (DOCX) [file pone.0264331.s002.docx]

**S3 Fig: Two clusters of acute symptoms and ongoing symptoms among these clusters**


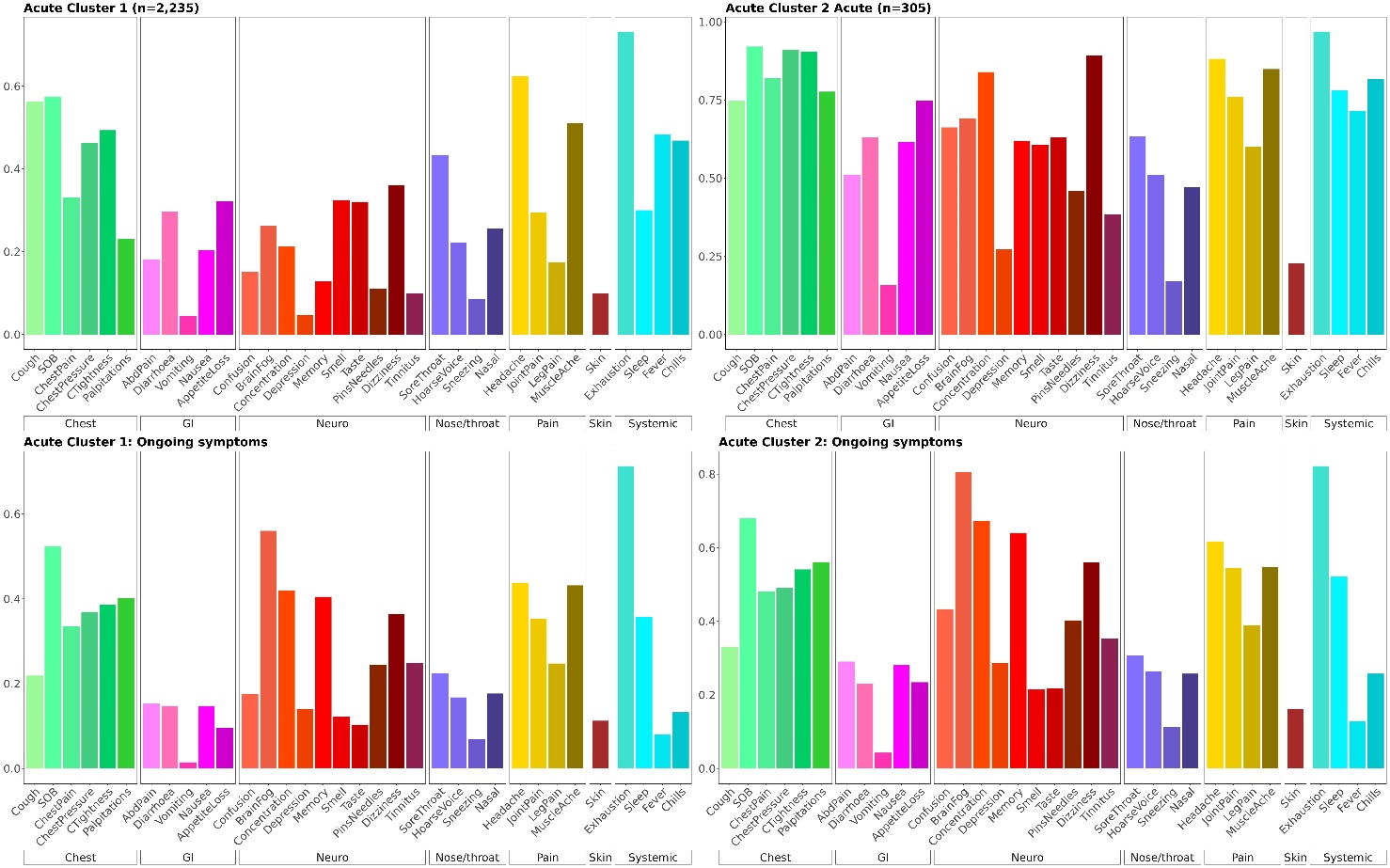

Supplement: S3 Fig — (DOCX) [file pone.0264331.s003.docx]

**S4 Fig: Clustering of lab confirmed subgroup only identifies similar clusters to whole dataset**


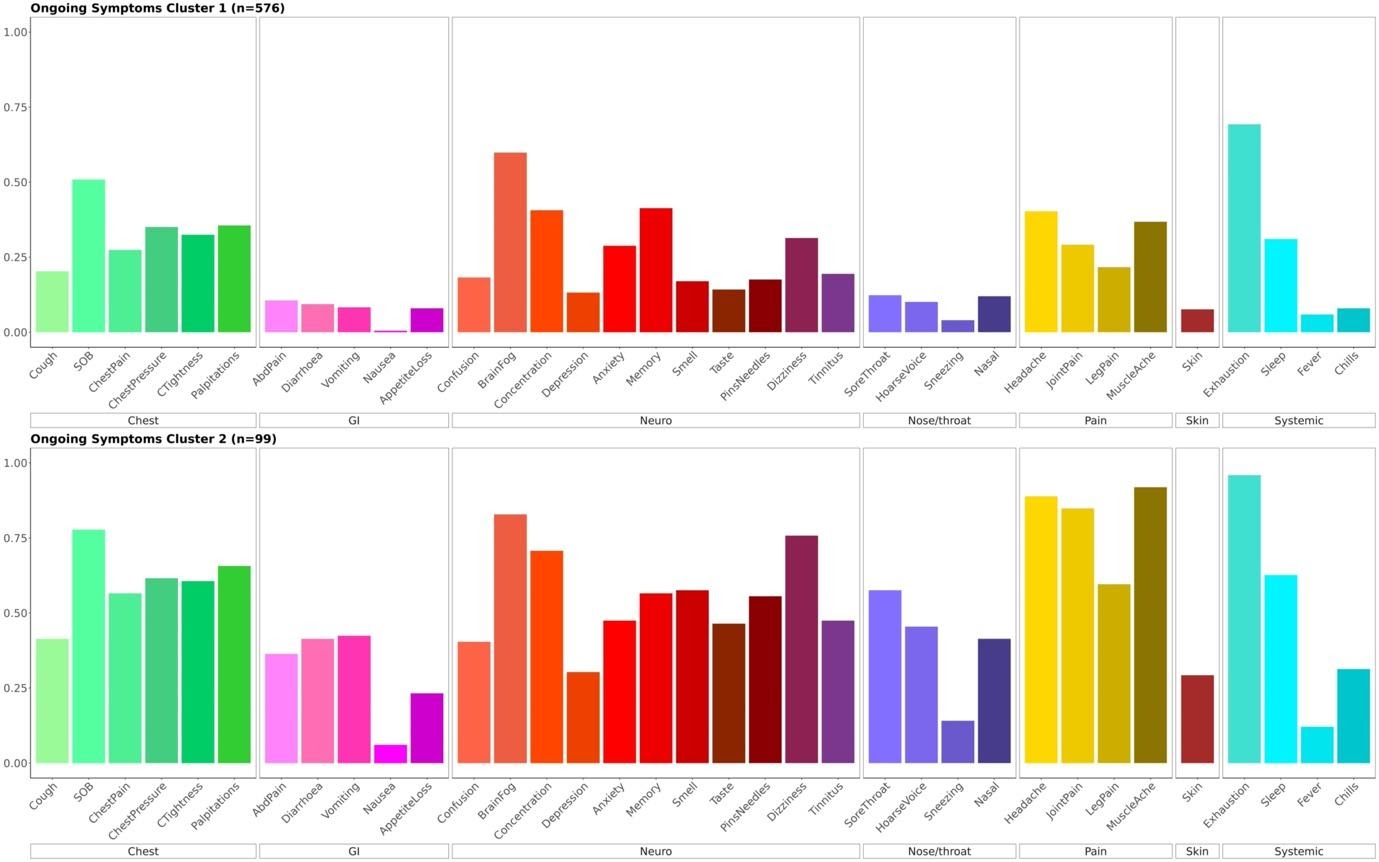

Supplement: S4 Fig — (DOCX) [file pone.0264331.s004.docx]
