# Supplementary material for: Characteristics and impact of Long Covid: Findings from an online survey
Source: PLoS One. 2022 Mar 8;17(3):e0264331. doi: 10.1371/journal.pone.0264331 (PMC8903286; doi:10.1371/journal.pone.0264331)

**S5 Fig: Transition from acute symptom clusters to ongoing symptom clusters by number of affected systems**


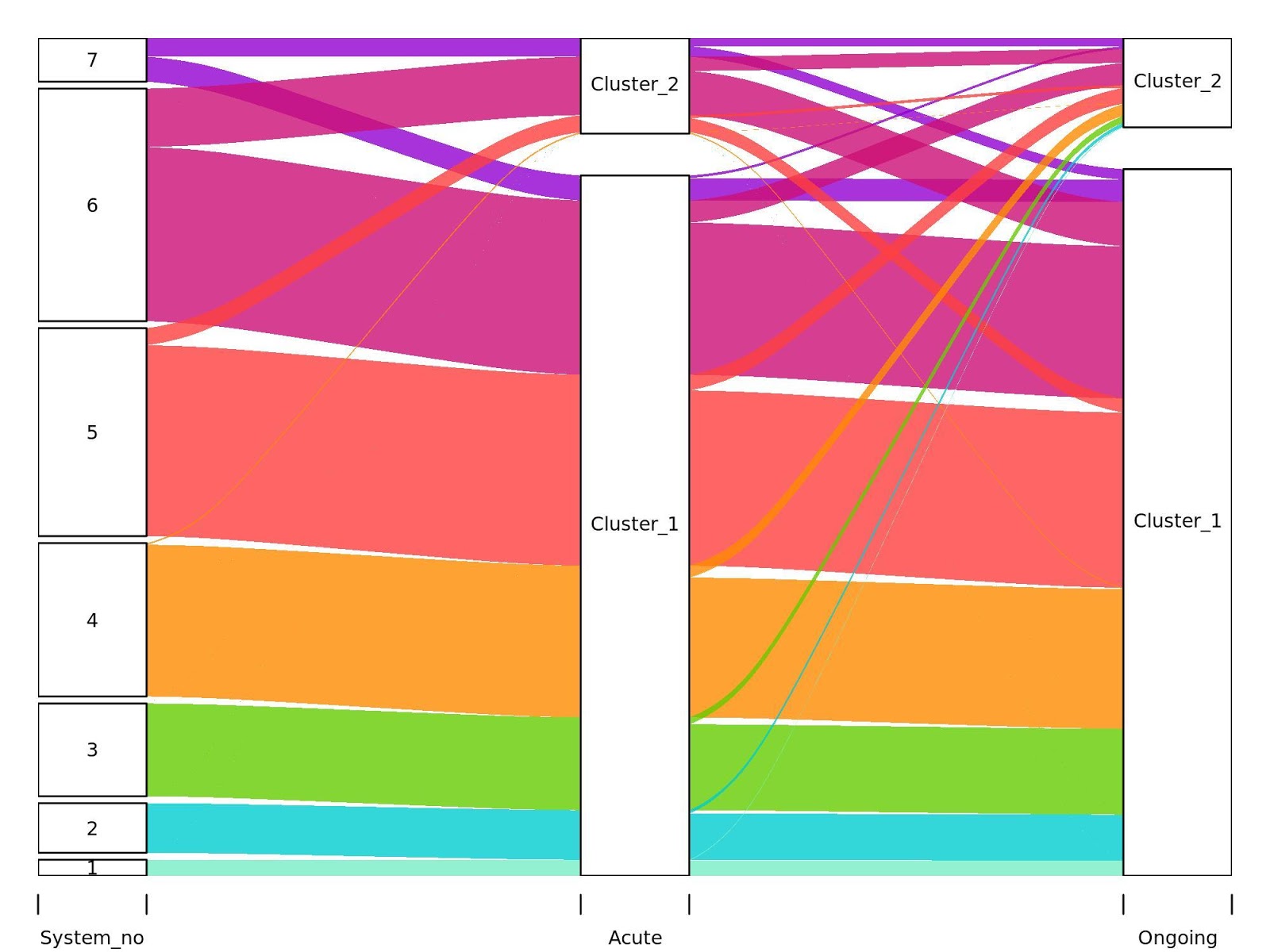

Supplement: S5 Fig — (DOCX) [file pone.0264331.s005.docx]
