# Supplementary material for: Characteristics and impact of Long Covid: Findings from an online survey
Source: PLoS One. 2022 Mar 8;17(3):e0264331. doi: 10.1371/journal.pone.0264331 (PMC8903286; doi:10.1371/journal.pone.0264331)

**S6 Fig: Mutually adjusted predictors of transition from acute symptom cluster 1 (ASC1: cardiopulmonary predominant) to ongoing symptom cluster 2 (OSC2: multisystem)**


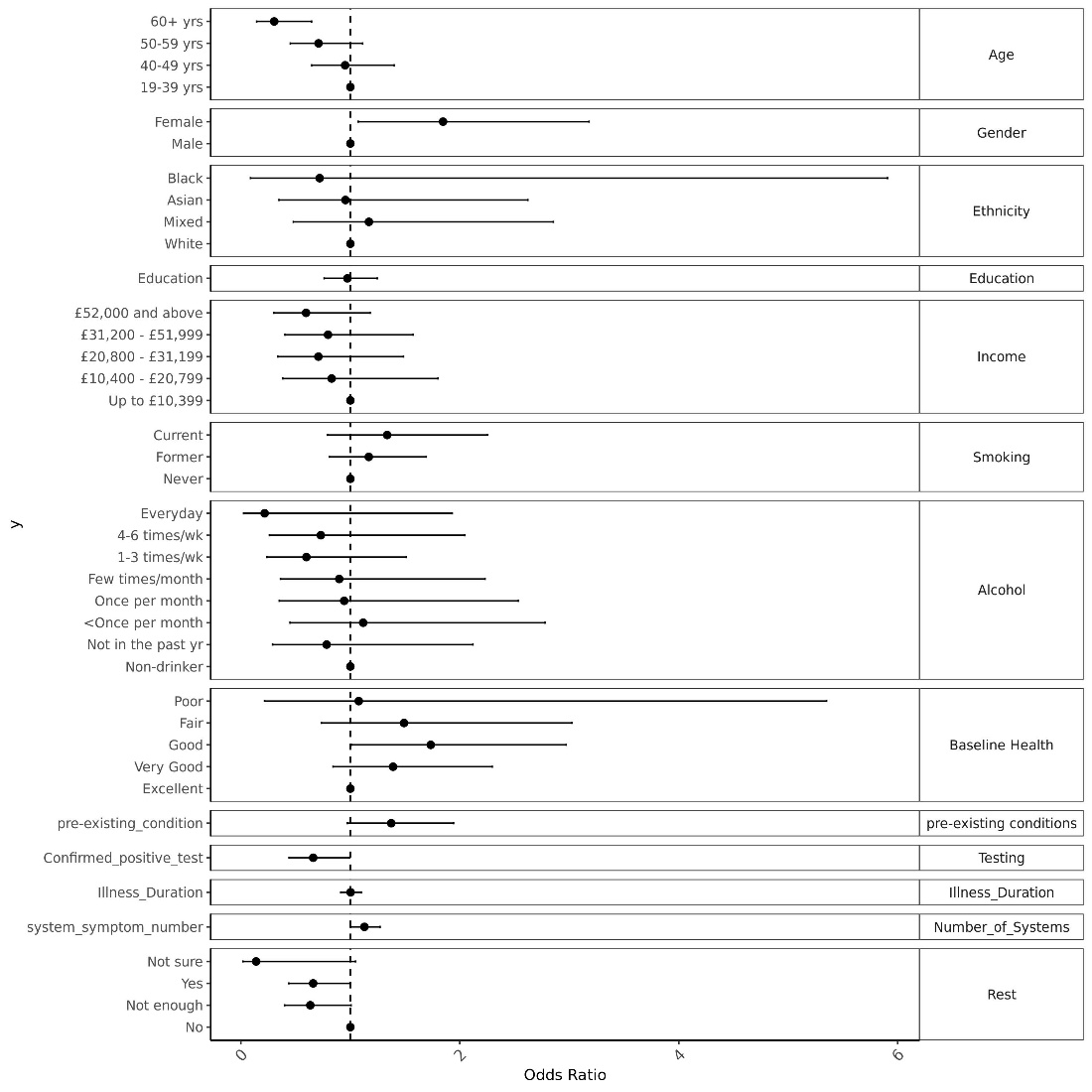

Supplement: S6 Fig — (DOCX) [file pone.0264331.s006.docx]
