# Supplementary material for: Characteristics and impact of Long Covid: Findings from an online survey
Source: PLoS One. 2022 Mar 8;17(3):e0264331. doi: 10.1371/journal.pone.0264331 (PMC8903286; doi:10.1371/journal.pone.0264331)
Supplement: S1 Table — (DOCX) [file pone.0264331.s007.docx]

**S1 Table: Classification of ongoing symptoms by organ system**

| **Ongoing symptom** | **Organ system** |
| --- | --- |
| Abdominal Pain | GI |
| Diarrhoea | GI |
| Vomiting | GI |
| Nausea | GI |
| Loss of appetite | GI |
| Cough | Cardiopulmonary |
| Shortness of breath | Cardiopulmonary |
| Chest pain | Cardiopulmonary |
| Chest pressure | Cardiopulmonary |
| Chest tightness | Cardiopulmonary |
| Palpitations | Cardiopulmonary |
| Confusion | Neuro |
| Brain fog | Neuro |
| Poor concentration | Neuro |
| Depression | Neuro |
| Anxiety | Neuro |
| Memory problems | Neuro |
| Altered or loss of sense of smell | Neuro |
| Altered or loss of sense of taste | Neuro |
| Pins and needles | Neuro |
| Dizziness | Neuro |
| Tinnitus | Neuro |
| Exhaustion | Systemic |
| Sleep disturbance | Systemic |
| Fever | Systemic |
| Chills | Systemic |
| Sore throat | Nose/throat |
| Hoarse voice | Nose/throat |
| Sneezing | Nose/throat |
| Nasal symptoms | Nose/throat |
| Headache | Pain |
| Joint pain | Pain |
| Leg pain | Pain |
| Muscle aches | Pain |
| Skin rash | Skin |
