# Supplementary material for: Characteristics and impact of Long Covid: Findings from an online survey
Source: PLoS One. 2022 Mar 8;17(3):e0264331. doi: 10.1371/journal.pone.0264331 (PMC8903286; doi:10.1371/journal.pone.0264331)
Supplement: S2 Table — (DOCX) [file pone.0264331.s008.docx]

**S2 Table: Pre-existing conditions in survey participants**

|  | Full sample | | Tested positive | | Tested negative or not tested | |
| --- | --- | --- | --- | --- | --- | --- |
|  | n | % | n | % | n | % |
|  | 2550 |  | 675 |  | 1793 |  |
| Allergies | 86 | 3.4 | 15 | 2.2 | 70 | 3.9 |
| Arthritis | 92 | 3.6 | 33 | 4.9 | 57 | 3.2 |
| Asthma | 344 | 13.6 | 99 | 14.7 | 240 | 13.5 |
| Cancer | 18 | 0.7 | 6 | 0.9 | 12 | 0.7 |
| Coeliac disease | 20 | 0.8 | 5 | 0.7 | 14 | 0.8 |
| Type 1 diabetes | 11 | 0.4 | 5 | 0.7 | 6 | 0.3 |
| Type 2 diabetes | 51 | 2.0 | 16 | 2.4 | 33 | 1.9 |
| Depression | 68 | 2.7 | 25 | 3.7 | 41 | 2.3 |
| Endometriosis | 29 | 1.2 | 5 | 0.7 | 24 | 1.3 |
| Epilepsy | 12 | 0.5 | 1 | 0.2 | 10 | 0.6 |
| Fibromyalgia | 61 | 2.4 | 18 | 2.7 | 41 | 2.3 |
| Hypertension | 141 | 5.6 | 42 | 6.3 | 88 | 4.9 |
| Hypothyroidism | 144 | 5.7 | 42 | 6.3 | 98 | 5.5 |
| Irritable bowel syndrome | 56 | 2.2 | 13 | 1.9 | 42 | 2.4 |
| Kidney disease | 12 | 0.5 | 2 | 0.3 | 10 | 0.6 |
| Liver disease | 18 | 0.7 | 4 | 0.6 | 14 | 0.8 |
| Migraine | 69 | 2.7 | 13 | 1.9 | 56 | 3.1 |
| Overweight/obesity | 38 | 1.5 | 14 | 2.1 | 24 | 1.3 |
| Polycystic ovary syndrome | 24 | 1.0 | 10 | 1.5 | 13 | 0.7 |
| Sleep disorders | 15 | 0.6 | 4 | 0.6 | 11 | 0.6 |
