# Supplementary material for: Characteristics and impact of Long Covid: Findings from an online survey
Source: PLoS One. 2022 Mar 8;17(3):e0264331. doi: 10.1371/journal.pone.0264331 (PMC8903286; doi:10.1371/journal.pone.0264331)
Supplement: S4 Table — (DOCX) [file pone.0264331.s010.docx]

**S4 Table: Duration and pattern of illness in those who reported full recovery from Long Covid**

|  | Recovered | | | | | |
| --- | --- | --- | --- | --- | --- | --- |
|  | Full sample | | Tested positive | | Test negative/not tested | |
|  | n | % | n | % | n | % |
|  | 58 |  | 14 |  | 41 |  |
| Duration of illness |  |  |  |  |  |  |
| ≥1-<2 months | 17 | 29.3 | 5 | 35.7 | 11 | 26.8 |
| ≥2-<3 months | 11 | 19.0 | 1 | 7.1 | 10 | 24.4 |
| ≥3-<4 months | 10 | 17.2 | 5 | 35.7 | 4 | 9.8 |
| ≥4-<5 months | 5 | 8.6 | - | - | 5 | 12.2 |
| ≥5-<6 months | 7 | 12.1 | - | - | 7 | 17.1 |
| ≥6-9 months | 8 | 13.8 | 3 | 21.4 | 4 | 9.8 |
| Time since last symptom |  |  |  |  |  |  |
| ≤2 weeks | 3 | 5.2 | 2 | 14.3 | 1 | 2.4 |
| >2weeks - <1 month | 8 | 13.8 | 1 | 7.1 | 6 | 14.6 |
| ≥1-<2 months | 12 | 20.7 | 3 | 21.4 | 9 | 22.0 |
| ≥2-<3 months | 12 | 20.7 | 4 | 28.6 | 7 | 17.1 |
| ≥3-<4 months | 7 | 12.1 | 3 | 21.4 | 4 | 9.8 |
| ≥4 months | 16 | 27.6 | 1 | 7.1 | 14 | 34.2 |
| Pattern of symptoms in last month of illness |  |  |  |  |  |  |
| Gradually got better | 25 | 43.1 | 6 | 42.9 | 17 | 41.5 |
| Fluctuating | 9 | 15.5 | 2 | 14.3 | 6 | 14.6 |
| Come and go | 24 | 41.4 | 6 | 42.9 | 18 | 43.9 |
