# Supplementary material for: Characteristics and impact of Long Covid: Findings from an online survey
Source: PLoS One. 2022 Mar 8;17(3):e0264331. doi: 10.1371/journal.pone.0264331 (PMC8903286; doi:10.1371/journal.pone.0264331)
Supplement: S5 Table — (DOCX) [file pone.0264331.s011.docx]

**S5 Table: Demographics and baseline health of survey participants**

|  | Full sample | | Tested positive | | Tested negative | | Not tested | | p-value^a^ |
| --- | --- | --- | --- | --- | --- | --- | --- | --- | --- |
|  | n | % | n | % | n | % | n | % |  |
| Total n | 2550 |  | 675 |  | 1247 |  | 546 |  |  |
| Age, years (mean ± SD) (n= 2543) | 46.5 ± 11.0 |  | 45.3 ± 10.9 |  | 45.9 ± 10.4 |  | 48.1 ± 11.5 |  | <0.001 |
| Age, categorised |  |  |  |  |  |  |  |  |  |
| 18-30 | 189 | 7.4 | 68 | 10.1 | 82 | 6.6 | 36 | 6.6 | <0.001 |
| 31-45 | 997 | 39.2 | 271 | 40.2 | 526 | 42.4 | 186 | 34.1 |  |
| 46-60 | 1051 | 41.4 | 275 | 40.8 | 506 | 40.7 | 235 | 43.1 |  |
| ≥60 | 305 | 12.0 | 60 | 8.9 | 128 | 10.3 | 88 | 16.2 |  |
| Gender (n=2547) |  |  |  |  |  |  |  |  |  |
| Male | 413 | 16.2 | 101 | 15.0 | 184 | 14.8 | 106 | 19.4 | 0.22 |
| Female | 2108 | 82.8 | 572 | 84.7 | 1050 | 84.3 | 427 | 78.2 |  |
| Non-binary | 21 | 0.8 | 1 | 0.2 | 10 | 0.8 | 10 | 1.8 |  |
| Prefer not to say | 3 | 0.1 | 1 | 0.2 | - | - | 2 | 0.4 |  |
| Other | 2 | 0.1 | - | - | 1 | 0.1 | 1 | 0.2 |  |
| Country (n=2523) |  |  |  |  |  |  |  |  |  |
| UK - England | 1665 | 66.0 | 410 | 61.5 | 846 | 68.3 | 357 | 65.7 | <0.001 |
| UK - Scotland | 215 | 8.5 | 34 | 5.1 | 91 | 7.4 | 85 | 15.7 |  |
| UK - Wales | 114 | 4.5 | 23 | 3.4 | 50 | 4.0 | 35 | 6.4 |  |
| UK - Northern Ireland | 22 | 0.9 | 6 | 0.9 | 10 | 0.8 | 5 | 0.9 |  |
| Outside the UK | 507 | 20.1 | 194 | 29.1 | 241 | 19.5 | 61 | 11.2 |  |
| Africa | 18 | 0.7 | 16 | 2.4 | 1 | 0.1 | 1 | 0.2 |  |
| Australia and New Zealand | 15 | 0.6 | 7 | 1.0 | 7 | 2.6 | 1 | 0.2 |  |
| Europe | 210 | 8.3 | 60 | 9.0 | 114 | 9.2 | 31 | 5.7 |  |
| South/Central America and Caribbean | 10 | 0.4 | 5 | 0.7 | 2 | 0.2 | 3 | 0.6 |  |
| North America | 232 | 9.2 | 93 | 13.9 | 113 | 9.1 | 20 | 3.7 |  |
| Asia | 15 | 0.6 | 7 | 1.0 | 4 | 0.3 | 4 | 0.7 |  |
| Middle East | 7 | 0.3 | 6 | 0.9 | - | - | 1 | 0.2 |  |
| Ethnicity (n=2533) |  |  |  |  |  |  |  |  |  |
| White | 2362 | 93.3 | 607 | 90.3 | 1173 | 94.2 | 515 | 94.7 | <0.001 |
| Mixed/Multiple ethnic backgrounds | 67 | 2.7 | 18 | 2.7 | 34 | 2.7 | 13 | 2.4 |  |
| Asian | 64 | 2.5 | 25 | 3.7 | 25 | 2.0 | 11 | 2.0 |  |
| Black/African/Caribbean | 23 | 0.9 | 15 | 2.2 | 7 | 0.6 | 1 | 0.2 |  |
| Other | 14 | 0.6 | 7 | 1.0 | 6 | 0.5 | 3 | 0.6 |  |
| Prefer not to say | 3 | 0.1 | - | - | - | - | 1 | 0.2 |  |
| Educational attainment (n=2527) |  |  |  |  |  |  |  |  |  |
| No formal qualifications | 37 | 1.5 | 11 | 1.7 | 14 | 1.1 | 10 | 1.9 | 0.31 |
| O levels or equivalent | 209 | 8.3 | 57 | 8.6 | 94 | 7.6 | 51 | 9.4 |  |
| A levels or equivalent | 331 | 13.1 | 79 | 11.8 | 155 | 12.5 | 81 | 14.9 |  |
| University degree or above | 1950 | 77.2 | 520 | 78.0 | 981 | 78.9 | 400 | 73.8 |  |
| Smoking status (n=2537) |  |  |  |  |  |  |  |  |  |
| Non-smoker | 1577 | 62.2 | 424 | 62.9 | 780 | 62.7 | 323 | 59.4 | 0.28 |
| Ex-smoker | 692 | 27.3 | 184 | 27.3 | 340 | 27.3 | 149 | 27.4 |  |
| Current smoker | 268 | 10.6 | 66 | 9.8 | 125 | 10.0 | 72 | 13.2 |  |
| Alcohol intake in the 12 months before COVID-19 (n=2539) |  |  |  |  |  |  |  |  |  |
| Do not drink | 91 | 3.6 | 35 | 5.2 | 40 | 3.2 | 14 | 2.6 | 0.008 |
| Did not drink in the past year | 254 | 10.0 | 50 | 7.4 | 123 | 9.9 | 69 | 12.7 |  |
| <Once a month | 452 | 17.8 | 137 | 20.3 | 206 | 16.5 | 100 | 18.4 |  |
| Once a month | 210 | 8.3 | 62 | 9.2 | 105 | 8.4 | 39 | 7.2 |  |
| Few times a month | 514 | 20.2 | 135 | 20.0 | 265 | 21.3 | 103 | 18.9 |  |
| 1-3 times a week | 708 | 27.9 | 186 | 27.6 | 359 | 28.8 | 139 | 25.5 |  |
| 4-6 times a week | 245 | 9.7 | 55 | 8.2 | 121 | 9.7 | 61 | 11.2 |  |
| Everyday | 65 | 2.6 | 14 | 2.1 | 27 | 2.2 | 20 | 3.7 |  |
| Baseline health before COVID-19 (n=2540) |  |  |  |  |  |  |  |  |  |
| Poor | 32 | 1.3 | 3 | 0.5 | 15 | 1.2 | 12 | 2.2 | 0.008 |
| Fair | 233 | 9.2 | 53 | 7.9 | 105 | 8.4 | 67 | 12.3 |  |
| Good | 675 | 26.6 | 199 | 29.5 | 316 | 25.3 | 146 | 26.8 |  |
| Very good | 1050 | 41.3 | 277 | 41.1 | 537 | 43.1 | 212 | 38.9 |  |
| Excellent | 550 | 21.7 | 142 | 21.1 | 274 | 22.0 | 108 | 19.8 |  |
| Pre-existing health conditions (n=2541) |  |  |  |  |  |  |  |  |  |
| No | 1339 | 52.7 | 337 | 49.9 | 667 | 53.5 | 298 | 54.6 | 0.21 |
| Yes | 1202 | 47.3 | 338 | 50.1 | 580 | 46.5 | 248 | 45.4 |  |

^a^Comparisons between those who tested positive, tested negative or were not tested for COVID-19 infection used ANOVA or Kruskal-Wallis test for continuous and chi square test for categorical variables.
