# Supplementary material for: Characteristics and impact of Long Covid: Findings from an online survey
Source: PLoS One. 2022 Mar 8;17(3):e0264331. doi: 10.1371/journal.pone.0264331 (PMC8903286; doi:10.1371/journal.pone.0264331)
Supplement: S6 Table — (DOCX) [file pone.0264331.s012.docx]

**S6 Table: Initial symptoms experienced at the start of COVID-19 illness (first two weeks)**

|  | Full sample | | Tested positive | | Tested negative | | Not tested | | p-value^a^ |
| --- | --- | --- | --- | --- | --- | --- | --- | --- | --- |
|  | n | % | n | % | n | % | n | % |  |
| n | 2540 |  | 675 |  | 1247 |  | 546 |  |  |
| Fever | 1298 | 51.1 | 362 | 53.6 | 600 | 48.1 | 293 | 53.7 | 0.02 |
| Cough | 1485 | 58.5 | 399 | 59.1 | 712 | 57.1 | 325 | 59.5 | 0.54 |
| Altered or loss of sense of smell | 922 | 36.3 | 410 | 60.7 | 293 | 23.5 | 194 | 35.5 | <0.001 |
| Altered or loss of sense of taste | 921 | 36.3 | 388 | 57.5 | 325 | 26.1 | 185 | 33.9 | <0.001 |
| Abdominal pain | 562 | 22.1 | 150 | 22.2 | 284 | 22.8 | 118 | 21.6 | 0.86 |
| Diarrhoea | 855 | 33.7 | 235 | 34.8 | 416 | 33.4 | 185 | 33.9 | 0.81 |
| Loss of appetite | 946 | 37.2 | 293 | 43.4 | 409 | 32.8 | 215 | 39.4 | <0.001 |
| Nausea | 642 | 25.3 | 176 | 26.1 | 319 | 25.6 | 132 | 24.2 | 0.74 |
| Vomiting | 148 | 5.8 | 47 | 7.0 | 70 | 5.6 | 29 | 5.3 | 0.39 |
| Cognitive dysfunction | 1168 | 46.0 | 315 | 46.7 | 554 | 44.4 | 268 | 49.1 | 0.18 |
| Brain fog | 797 | 31.4 | 226 | 33.5 | 364 | 29.2 | 186 | 34.1 | 0.05 |
| Confusion | 539 | 21.2 | 137 | 20.3 | 258 | 20.7 | 127 | 23.3 | 0.39 |
| Memory problems | 475 | 18.7 | 152 | 22.5 | 210 | 16.8 | 101 | 18.5 | 0.01 |
| Poor concentration | 730 | 28.7 | 198 | 29.3 | 346 | 27.8 | 170 | 31.1 | 0.33 |
| Depression | 187 | 7.4 | 57 | 8.4 | 84 | 6.7 | 42 | 7.7 | 0.38 |
| Chest pain | 991 | 39.0 | 239 | 35.4 | 523 | 41.9 | 205 | 37.6 | 0.01 |
| Chest pressure | 1314 | 51.7 | 323 | 47.9 | 688 | 55.2 | 279 | 51.1 | 0.007 |
| Chest tightness | 1379 | 54.3 | 338 | 50.1 | 723 | 58.0 | 293 | 53.7 | 0.003 |
| Palpitations | 754 | 29.7 | 215 | 31.9 | 367 | 29.4 | 154 | 28.2 | 0.35 |
| Shortness of breath | 1566 | 61.7 | 405 | 60.0 | 786 | 63.0 | 335 | 61.4 | 0.41 |
| Chills | 1296 | 51.0 | 359 | 53.2 | 626 | 50.2 | 284 | 52.0 | 0.44 |
| Dizziness | 1079 | 42.5 | 304 | 45.0 | 501 | 40.2 | 237 | 43.3 | 0.10 |
| Exhaustion | 1928 | 75.9 | 514 | 76.2 | 940 | 75.4 | 427 | 78.2 | 0.43 |
| Headache | 1663 | 65.5 | 480 | 71.1 | 811 | 65.0 | 327 | 60.0 | <0.001 |
| Hoarse voice | 653 | 25.7 | 156 | 23.1 | 345 | 27.8 | 137 | 25.1 | 0.08 |
| Nasal symptoms | 717 | 28.2 | 231 | 34.2 | 313 | 25.1 | 153 | 28.0 | <0.001 |
| Sore throat | 1161 | 45.7 | 291 | 43.1 | 589 | 47.2 | 248 | 45.4 | 0.22 |
| Sneezing | 242 | 9.5 | 85 | 12.6 | 108 | 8.7 | 40 | 7.3 | 0.003 |
| Tinnitus | 339 | 13.4 | 104 | 15.4 | 150 | 12.0 | 67 | 12.3 | 0.09 |
| Joint pain | 890 | 35.0 | 290 | 43.0 | 395 | 30.9 | 199 | 36.5 | <0.001 |
| Leg pain | 573 | 22.6 | 179 | 26.5 | 249 | 20.0 | 121 | 22.2 | 0.004 |
| Muscle aches | 1402 | 55.2 | 425 | 63.0 | 653 | 52.4 | 283 | 51.8 | <0.001 |
| Pins and needles | 388 | 15.3 | 109 | 16.2 | 187 | 15.0 | 76 | 13.9 | 0.55 |
| Skin rash | 289 | 11.4 | 81 | 12.0 | 141 | 11.3 | 57 | 10.4 | 0.69 |
| Sleep disturbance | 909 | 35.8 | 243 | 36.0 | 426 | 34.2 | 212 | 38.8 | 0.16 |
| Number of initial symptoms, mean ± SD, median (interquartile range) | 12 ± 6  11 (7 to 16) |  | 13 ± 6  12 (8 to 17) |  | 11 ± 6  10 (7 to 15) |  | 12 ± 6  11 (7 to 16) |  | <0.001 |

^a^Comparisons between those who tested positive, tested negative or were not tested for COVID-19 infection used ANOVA or Kruskal-Wallis test for continuous and chi square test for categorical variables.
