# Supplementary material for: Characteristics and impact of Long Covid: Findings from an online survey
Source: PLoS One. 2022 Mar 8;17(3):e0264331. doi: 10.1371/journal.pone.0264331 (PMC8903286; doi:10.1371/journal.pone.0264331)
Supplement: S7 Table — (DOCX) [file pone.0264331.s013.docx]

**S7 Table: Ongoing symptoms, fatigue severity and organ systems affected**

|  | Full sample | | Tested positive | | Tested negative | | Not tested | | p-value^a^ |
| --- | --- | --- | --- | --- | --- | --- | --- | --- | --- |
|  | n | % | n | % | n | % | n | % |  |
| n | 2526 |  | 675 |  | 1247 |  | 546 |  |  |
| Ongoing symptoms |  |  |  |  |  |  |  |  |  |
| Fever | 217 | 8.6 | 46 | 6.8 | 136 | 10.9 | 31 | 5.7 | <0.001 |
| Cough | 587 | 23.3 | 158 | 23.4 | 315 | 25.3 | 98 | 18.0 | 0.003 |
| Altered or loss of sense of smell | 358 | 14.2 | 165 | 24.4 | 118 | 9.5 | 65 | 11.9 | <0.001 |
| Altered or loss of sense of taste | 313 | 12.4 | 141 | 20.9 | 110 | 8.8 | 54 | 9.9 | <0.001 |
| Abdominal pain | 427 | 16.9 | 97 | 14.4 | 245 | 19.7 | 74 | 13.6 | 0.001 |
| Diarrhoea | 398 | 15.8 | 95 | 14.1 | 218 | 17.5 | 75 | 13.8 | 0.05 |
| Loss of appetite | 283 | 11.2 | 69 | 10.2 | 158 | 12.7 | 52 | 9.5 | 0.09 |
| Nausea | 412 | 16.3 | 90 | 13.3 | 246 | 19.7 | 69 | 12.7 | <0.001 |
| Vomiting | 46 | 1.8 | 9 | 1.3 | 30 | 2.41 | 7 | 1.3 | 0.13 |
| Anxiety | 715 | 28.3 | 213 | 31.6 | 349 | 28.0 | 144 | 26.4 | 0.11 |
| Cognitive dysfunction | 1747 | 69.2 | 480 | 71.1 | 885 | 71.0 | 347 | 63.7 | 0.005 |
| Brain fog | 1490 | 59.0 | 427 | 63.3 | 741 | 59.4 | 293 | 53.8 | 0.004 |
| Confusion | 520 | 20.6 | 145 | 21.5 | 267 | 21.4 | 96 | 17.6 | 0.15 |
| Memory problems | 1094 | 43.3 | 294 | 43.6 | 558 | 44.8 | 219 | 40.2 | 0.20 |
| Poor concentration | 1138 | 45.1 | 304 | 45.0 | 594 | 47.6 | 220 | 40.4 | 0.02 |
| Depression | 397 | 15.7 | 106 | 15.7 | 191 | 15.3 | 92 | 16.9 | 0.70 |
| Chest pain | 891 | 35.3 | 214 | 31.7 | 501 | 40.2 | 155 | 28.4 | <0.001 |
| Chest pressure | 970 | 38.4 | 263 | 39.0 | 525 | 42.1 | 170 | 31.2 | <0.001 |
| Chest tightness | 1023 | 40.5 | 247 | 36.6 | 574 | 46.0 | 178 | 32.7 | <0.001 |
| Palpitations | 1062 | 42.0 | 270 | 40.0 | 576 | 46.2 | 198 | 36.3 | <0.001 |
| Shortness of breath | 1370 | 54.2 | 370 | 54.8 | 711 | 57.0 | 266 | 48.8 | 0.006 |
| Chills | 373 | 14.8 | 77 | 11.4 | 212 | 17.0 | 74 | 13.6 | 0.003 |
| Dizziness | 980 | 38.8 | 256 | 37.9 | 524 | 42.0 | 179 | 32.8 | 0.001 |
| Exhaustion | 1834 | 72.6 | 494 | 73.2 | 920 | 73.8 | 378 | 69.4 | 0.15 |
| Headache | 1161 | 46.0 | 320 | 47.4 | 631 | 50.6 | 196 | 36.0 | <0.001 |
| Hoarse voice | 453 | 17.9 | 103 | 15.3 | 266 | 21.3 | 76 | 13.9 | <0.001 |
| Nasal symptoms | 471 | 18.7 | 110 | 16.3 | 264 | 21.2 | 89 | 16.3 | 0.008 |
| Sore throat | 591 | 23.4 | 128 | 19.0 | 343 | 27.5 | 111 | 20.4 | <0.001 |
| Sneezing | 188 | 7.4 | 37 | 5.5 | 108 | 8.7 | 38 | 7.0 | 0.04 |
| Tinnitus | 662 | 26.2 | 159 | 23.6 | 359 | 28.8 | 118 | 21.7 | 0.002 |
| Joint pain | 950 | 37.6 | 252 | 37.3 | 496 | 39.8 | 185 | 33.9 | 0.06 |
| Leg pain | 668 | 26.4 | 184 | 27.3 | 339 | 27.2 | 133 | 24.4 | 0.42 |
| Muscle aches | 1126 | 44.6 | 303 | 44.9 | 572 | 45.9 | 223 | 40.9 | 0.15 |
| Pins and needles | 667 | 26.4 | 156 | 23.1 | 386 | 31.0 | 115 | 21.1 | <0.001 |
| Skin rash | 299 | 11.8 | 73 | 10.8 | 165 | 13.2 | 52 | 9.5 | 0.06 |
| Sleep disturbance | 952 | 37.7 | 241 | 35.7 | 504 | 40.4 | 187 | 34.3 | 0.02 |
| Number of ongoing symptoms, mean ± SD, median (interquartile range) | 10 ± 6  9 (5 to 14) | | 10 ± 6  9 (5 to 13) | | 11 ± 6  10 (6 to 15) | | 9 ± 6  8 (4 to 12) | | 0.49 |
| Fatigue Severity Scale score, mean ± SD (n=2000) | 5.5 ± 1.4 | | 5.5 ± 1.4 | | 5.6 ± 1.4 | | 5.4 ± 1.4 | | 0.11 |
| Score ≥4 % | 86 | | 84 | | 87 | | 83 | |  |
| Number of organ systems affected |  |  |  |  |  |  |  |  |  |
| 1 | 121 | 4.8 | 29 | 4.3 | 45 | 3.6 | 46 | 8.4 | <0.001 |
| 2 | 253 | 10.0 | 81 | 12.0 | 102 | 8.2 | 62 | 11.4 |  |
| 3 | 437 | 17.3 | 120 | 17.8 | 210 | 16.8 | 98 | 18.0 |  |
| 4 | 623 | 24.7 | 185 | 27.4 | 292 | 23.4 | 129 | 23.7 |  |
| 5 | 551 | 21.8 | 145 | 21.5 | 278 | 22.3 | 115 | 21.1 |  |
| 6 | 380 | 15.0 | 77 | 11.4 | 234 | 18.8 | 61 | 11.2 |  |
| 7 | 119 | 4.7 | 29 | 4.3 | 70 | 5.6 | 18 | 3.3 |  |
| Organ systems affected by symptoms |  |  |  |  |  |  |  |  |  |
| Gastrointestinal | 909 | 36.0 | 220 | 32.6 | 500 | 40.1 | 166 | 30.5 | <0.001 |
| Chest (cardiopulmonary) | 2070 | 82.0 | 552 | 81.8 | 1055 | 84.6 | 416 | 76.3 | <0.001 |
| Neurological | 2164 | 85.7 | 582 | 86.2 | 1091 | 87.5 | 439 | 80.6 | 0.001 |
| Systemic | 2035 | 80.6 | 541 | 80.2 | 1023 | 82.0 | 422 | 77.4 | 0.07 |
| Nose/Throat | 1036 | 41.0 | 232 | 34.4 | 584 | 46.8 | 204 | 37.4 | <0.001 |
| Pain | 1785 | 70.7 | 481 | 71.3 | 913 | 73.2 | 348 | 63.9 | <0.001 |
| Skin | 299 | 11.8 | 73 | 10.8 | 165 | 13.2 | 52 | 9.5 | 0.06 |

^a^Comparisons between those who tested positive, tested negative or were not tested for COVID-19 infection used ANOVA or Kruskal-Wallis test for continuous and chi square test for categorical variables.
