# Supplementary material for: Characteristics and impact of Long Covid: Findings from an online survey
Source: PLoS One. 2022 Mar 8;17(3):e0264331. doi: 10.1371/journal.pone.0264331 (PMC8903286; doi:10.1371/journal.pone.0264331)
Supplement: Attachment — Submitted filename: Response_to_reviewers_LC final 17 12 21.docx [file pone.0264331.s014.docx]

ACADEMIC EDITOR:

*Thank you for the helpful feedback which we address point by point below. Since it has been a long while since we submitted this manuscript to PLOS One, we have also gone through the whole paper and updated information and references when necessary. We have changed some of the preprints referenced to their current journal citations if relevant. The original referencing and text remains in the preprint version of our paper:*
<https://www.medrxiv.org/content/10.1101/2021.03.21.21253968v2.full.pdf>

The reviewers raise important issues. While Reviewer #1 had concerns about the data that the authors cannot address and already acknowledge in the discussion, please do the following to address comments #1-3:

- expand the discussion of the non-representativeness of the sample to contrast it with the demographic characteristics of cases in the UK at the time

*Thank you, we have expanded the discussion comparing to an earlier release from the UK’s Office for National Statistics on the prevalence of Long Covid (lines 475-479 in the clean and 494-498 in the tracked version):*

*“Data from the Office from National Statistics’ Coronavirus Infection Survey, a large randomly-sampled survey of around 140,000 households from all parts of the UK, showed higher prevalence of Long Covid following confirmed or suspected infection in women, adults aged 35 to 69 years, and those living in most deprived areas, with no stark differences between ethnic groups^28,29^”.*

- acknowledge that those with more symptoms and more severe symptoms may be more likely to respond

*Thank you, we have now added this sentence in the discussion (lines 490-491 in the clean and 516-517 in the tracked version).*

- the lack of lab confirmation is discussed and justified but is still concerning. Is there any data on what else was circulating at the time (e.g. influenza) in the UK that could be added to the discussion to feel more confident that these were COVID cases? It would also be helpful to see separate out test negatives and not tested for Tables 1-3 to see if the not tested were more similar to the test positives or negatives - these can be added as supplemental tables.

*Thank you for the suggestion, we have now included the test negative/not tested split in the supplementary tables 5-7. We believe there are three factors that are essential to consider here: the timing and availability of covid testing, and the potential for the pathological processes involved in Long Covid to influence the direction of the test result.*

*As we state in the results section: “The date of PCR test was available for 1491 of these 1582 participants. Twenty percent (n=304) were first tested at 0-5 days of onset of symptoms, with 72.7% of this group testing positive, while 80% (n=1187) were first tested ≥6 days of onset of symptoms, with 12.6% of this group testing positive.” We know that the sensitivity of PCR declines with time from onset of infection, decreasing from 77% at 4 days to 50% by 10 days (Hellewell J, Russell TW, Matthews R, Severn A, Adam S, Enfield L, et al. Estimating the effectiveness of routine asymptomatic PCR testing at different frequencies for the detection of SARS-CoV-2 infections. BMC Medicine. 2021 Apr 27;19(1):106).*

*With regards to the availability of testing, 73% of respondents reported first developing symptoms before June 2020 in the UK when community testing was not available for those not admitted to hospital or not in health/social care. We touch on this in the discussion under the ‘Patient involvement’ subheading, and actually consider the inclusion of those with suspected infection a strength of the study: “Although we list including suspected as well as lab-confirmed cases in the survey as a limitation, we also consider this a strength of the survey. Community testing in the UK was stopped in early March 2020 but became available to essential workers in May 2020, and community testing was restarted in late Spring/Summer 2020^24^. It is vital that people who got infected in the first wave of the pandemic and unable to access testing during the acute phase of their illness are included in research. They represent a big proportion of people currently living with Long Covid, and have the longest duration of illness, making it essential for studies about disease progression and prognosis to include them. We argue that those experiencing acute COVID-19 symptoms at a time of high national prevalence of infection, and not recovered for months after their acute episode, are very likely to have been infected with SARS-CoV-2 even if their access to lab testing was delayed or not possible at the time.” The senior author has written extensively, solely and with others, on the potential serious inequalities in recognition and support resulting from that unavailability of testing at the start of the pandemic and from only including those who have evidence of a positive test in Long Covid care, surveillance, and research, for example:*

*Alwan NA. A negative COVID-19 test does not mean recovery. Nature. 2020 Aug 11;584(7820):170–170.*

*Alwan NA. The road to addressing Long Covid. Science. 2021 Jul 30;373(6554):491–3.*

*Alwan NA, Attree E, Blair JM, Bogaert D, Bowen M-A, Boyle J, et al. From doctors as patients: a manifesto for tackling persisting symptoms of covid-19. BMJ. 2020 Sep 15;370:m3565.*

*Alwan NA. The teachings of Long COVID. Commun Med. 2021 Jul 12;1(1):1–3.*

*Alwan NA, Johnson L. Defining long COVID: Going back to the start. Med. 2021 Mar 25;2(5):501–4.*

*We consider it very important for research published in medical journals, which plays a core role in shaping clinical pathways and agendas, to factor in this inequality angle.*

*The third point is specific to antibody testing. There is emerging evidence that Long Covid is in itself linked to weak antibody response to SARS-CoV-2 and this could be implicated in the immunological process underlying this disease. We have cited a reference to support this claim (lines 505-507 in the clean and 531-533 in the tracked version): García-Abellán J, Padilla S, Fernández-González M, García JA, Agulló V, Andreo M, et al. Antibody Response to SARS-CoV-2 is Associated with Long-term Clinical Outcome in Patients with COVID-19: a Longitudinal Study. J Clin Immunol. 2021 Oct 1;41(7):1490–501.*

Please address or respond to all other comments from reviewer #2 and #3.

Additional minor comments to be addressed:

- healthcare utilization - please ensure to use language that is not specific to the UK or provide an explanation (e.g. 111 calls - are these emergency calls?)

*111 is a non-emergency medical care number that guides callers to the right service. The number is specific to the UK. We have deleted reference to 111 calls and updated this to “calls to non-emergency medical care number” (lines 346-347 in the clean and 364-365 in the tracked version).*

- Figure 2 - I assumed that the x-axis groups (reasons) were the strata but in fact the series groups (work pattern) are the strata and add up to 100% - consider switching

*Thank you, we have switched the strata as suggested.*

- add sample sizes to supplementary figure 1 so that it can be matched up with the text in the results

*Thank you, we have added sample sizes to supplementary figure 1.*

- unclear how Supplementary Figure 3 and Figure 3 are different

*Figure 3 presents the acute (bottom panels) and ongoing (top panels) symptoms by the two ongoing symptom clusters. Supplementary Figure 3 presents the same by the two acute symptom clusters.*

- Review titles for each panel in figure 3 as they all include 'ongoing' and are hard to match up with the groups. Add the acronyms used in the text (ASC1/ASC2/OSC1/OSC2).

*Thank you, we have the appropriate acronyms used in the text (OSC1/OSC2). The top panels present ongoing symptoms in the ongoing clusters and the bottom two panels present acute symptoms in the ongoing clusters.*

- Discussion - the authors mention recall bias as it relates to the acute stage, but this also applies to functional status at 6 weeks as most participants are far beyond 6 weeks of illness and should be mentioned.

*Thank you, we have added this to the sentence on recall bias (line 485 in the clean and 511 in the tracked version).*

==============================

Reviewers' comments:

**Reviewer #2:**

General: Although this survey appears to be nearly a year old, the overall topic remains quite timely and of great relevance. This article is a nice addition to the Long COVID literature in that it includes a rich dataset and wide array of analyses. Further clarifying the approach in the abstract, more clearly presenting the comparison between lab-confirmed and not in the results, and better describing the trends in symptoms over time would strengthen this article.

Abstract:
The main manuscript does a great job laying out the approach, methods, results, and limitations and is also much clearer about the engagement of the pwLC in the process and the recruitment methods. The abstract is much harder to follow. It would help to be more overt that the study specifically targeted populations who already identified themselves as having Long COVID. The methods should include the basic statistical analysis) (descriptive, comparison between lab-confirmed and not) in addition to the approach to clustering as the former makes up a good portion of the paper. The flow of the results could be improved. It would help to know much earlier that only 26.5% of the participants were lab confirmed (would more closely mirror the flow in the manuscript). Rather than use the term “Biggest difference” it would be good to clarify if this is the only statistically significant difference in reported symptoms between these two groups and what was the rate that this was reported in each group. Please clarify the difference between fluctuating and relapsing in the manuscript.

*Thank you, we have updated the abstract as suggested. We have added subheadings, amended the methods as suggested above, and moved the percentage lab confirmed up as suggested.*

*There are several statistically significant differences between the two groups. We highlighted loss of sense of smell/taste as the reported prevalence as an ongoing symptom in those that tested positive (24.4% smell, 20.9% taste) is over double that in those that tested negative/not tested (10.2% smell, 9.2% taste) which is statistically significant but also a big difference between the groups. For other statistically significant differences between symptoms, the difference between the groups was usually around 10% or less. These are listed in table 3 and include: sore throat, abdominal pain, nausea, brain fog, chest pain, chest tightness, chills, hoarse voice and a feeling of pins and needles. We have added these to the text of the results section (lines 258-262 in the clean and 282-286 in the tracked version), as well as differences between the groups in acute symptoms too (lines 266-270 in the clean and 272-277 in the tracked version) but have removed reference to differences from the abstract to fit in within the word count.*

*We have clarified the difference between fluctuating (intensity of symptoms changes but symptoms never completely go away) and relapsing (experience symptom-free periods in between relapses) in the results section of the manuscript (lines 293-295 in the clean and 310-312 in the tracked version).*

Introduction:
Potentially of interest, just this week the WHO released a case definition of post COVID conditions – you may want to consider citing this

*Thank you for highlighting this. The senior author was part of the WHO clinical case definition working group as cited in the report. The WHO definition document is now cited in the last paragraph of the discussion (line 673): A clinical case definition of post COVID-19 condition by a Delphi consensus, 6 October 2021 [Internet]. [cited 2021 Oct 15]. Available from:* [*https://www.who.int/publications-detail-redirect/WHO-2019-nCoV-Post_COVID-19_condition-Clinical_case_definition-2021.1*](https://www.who.int/publications-detail-redirect/WHO-2019-nCoV-Post_COVID-19_condition-Clinical_case_definition-2021.1)

Pg 4- It sounds like the initial symptoms are meant to be initial symptoms of COVID-19 and then ongoing symptoms are ongoing symptoms of Long COVID. Please confirm. Please also more explicitly define “ongoing” somewhere. Is that >4 weeks or ongoing at the time of the survey?

*Thank you. Initial symptoms are symptoms experienced in the first two weeks of infection. Ongoing symptoms are symptoms that remained or developed after the acute stage (≥2 weeks) and continue to be experienced in the longer term over the course of the illness. We have clarified this in the methods section (lines 151-154 in the clean and 164-167 in the tracked version).*

Methods:
A fair amount of language in this section seems more appropriate for the discussion section.

*Thank you. If the reviewer is referring to the description of how the survey was co-produced with patients, we feel this needs to stay in the methods section.*

Pg 5 – It would be good to more clearly use the term “Self-reported” for symptoms and diagnosis, both here and in the abstract

*We have added the term ‘self-reported’ to the abstract (line 28 in the clean and 33-34 in the tracked version). Symptoms are by definition self-reported as there is no other way to measure them.*

Results:
In the methods, you noted that comparison was made between lab-confirmed and not – if there is space, it would help to have that comparison included in each section. (Could consider reducing the length of the discussion section some to allow for this)

*Thank you, we have added comparison in each section as suggested (lines 253-255, 272-289, 306-309, 313-316, 323-234, 333-334, 342-344 and 355-356, all in the tracked version).*

You also note a large number of persons who tested negative, specifically comparing the positive and negative groups and assessing for differences there would also be of interest (though acknowledging that is a lot more work, I would see that as optional and just encourage the team to look at this)

Table 1 - Would recommend splitting "Tested negative" and "not tested" if possible

*Thank you for the suggestion, we have split the tested negative/not tested group and presented in the supplemental tables (5-7). We have chosen to retain the tables as is in the main analysis. Please see our response above to a similar comment by the editor/reviewer 1.*

Pg 7 - Please clarify is the median duration of illness, was median time since illness, or median duration of symptoms

*Median duration of illness is the duration of experiencing symptoms. We have added this in brackets to clarify (line 233 in the clean and 247 in the tracked version).*

Please clarify if “ongoing’ symptoms in the methods and results indicates at >4 wks or at the time of the survey. It would be helpful to include an overall overview of how symptoms decreased over time if they did this, such as xx (%) persons reported one or more ongoing symptoms for > 4weeks, xx (%) for > 12-weeks, and xx (%) for > 6 months following their initial infection.

*Ongoing symptoms are symptoms experienced in the longer-term over the course of the illness which could be anytime past the first 2 weeks (lines 153-154 in the clean and 174-175 in the tracked version). We did not collect data on the duration that each symptom was experienced for so cannot include the suggested overview.*

Pg 11 – for two clusters, it might help to identify the most commonly reported symptoms in each cluster in the manuscript
*Thank you, we have added the most commonly reported symptoms in the manuscript (lines 383-389 and 395-402 in the clean, and 401-407 and 414-421 in the tracked version).*

Discussion:
The summary of evolution of symptoms over time at the end of the first paragraph was helpful and not as clear in the results section. In general this section is quite verbose and could likely be written more concisely in order to allow for some of the recommendations above.
*Thank you, we have cut down this section in several places where we felt possible/appropriate.*

Table 3 - There are a number of symptoms listed here, for which there appears to be a statistically significant difference in frequency between those who tested positive and those who did not, that weren't highlighted in the manuscript. Would recommend more explicitly including/listing these as well.

*Thank you, we have highlighted these differences in the manuscript. Please see response to editors/other reviewers on this above (lines 262-270 in the clean and 277-286 in the tracked version).*

**Reviewer #3:**

The authors analyze data generated from a cross-sectional, online survey exploring long Covid features in subjects from the UK. The paper has significant amount of material, and the study is timely, and relevant. My comments are as follows:

(a) The purpose of the Abstract will be better served if re-written as Background, Methods, Results, and Conclusions. Otherwise, the long writeup doesn't appear very pleasing to go through.
*Thank you, we have added the suggested sections to the abstract.*

(b) Statistical analysis: t-tests were used for continuous variables. How was the assumption of Normality checked? Under violations, better to resort to 2-sample Wilcoxon tests.
*Thank you, we used t-tests or Mann Whitney U test for continuous variables. We had added this as a footnote to the tables where relevant (Tables 2, 3 and 6) but had not updated the methods section. We have now added this to the methods (line 181 in the clean and 194 in the tracked version).*

(c) Statistical analysis: Hierarchical agglomerative clustering (HAC) was used, utilizing the complete method. More details are needed on what that is. A variety of other methods, such as Ward, single-linkage, etc are available. Why were they not used? Justify.

*We used multiple methods for clustering in our early analysis. We examined K-medoids clustering, and Gaussian mixture model clustering (to allow for non-spherical clusters). We assessed the optimal number of clusters using both these methods with the silhouette method (for K-medoids) and Bayesian information criteria (BIC) for Guassian mixture clustering, and the stability of clusters using Jaccard’s coefficient. While K-medoids clustering identified two clusters, the Jaccard’s coefficient was below 0.75 suggesting these clusters were unstable. With Gaussian mixture clustering, we evaluated BIC for up to 15 clusters. However, BIC continued to increase with the number of clusters, suggesting a very high number of clusters would be optimal. Given the instability of clusters, and the need for better interpretability of clusters, we chose to carry out exploratory clustering using hierarchical agglomerative clustering rather than apply methods with unstable clusters, or potentially very large numbers of clusters that would hamper interpretability. We consider these methods exploratory rather than an exhaustive evaluation of clustering of these data. Given different approaches to HAC can lead to different clusters, we used the complete method, average method, Ward’s method, and single method. Using these, we noted that the single method was not optimal as it led to single individuals being members of clusters. Among other methods, the correlation between all methods was moderate to high but this was highest between the complete method and the average method (r=0.7), so we chose the complete method for our exploratory analysis. We note that our clusters in addition to correlating strongly with functional indicators, also accord well with the clusters obtained from other studies such as REACT-1 providing evidence for their clinical significance, and consistency with other studies.*

(d) Statistical analysis: HAC do not work for missing data, and can be quite sensitive to the choice of the distance/dissimilarity matrix employed. A sensitivity analysis, however small, would be very relevant here. If not, justification is needed behind the choice of the specific dissimilarity matrix used.

*We note that there was very little missing data in our survey. While we carried out a complete data analysis, we note that only 10 individuals (less than 0.4% of our sample was excluded due to missing data). We do not consider that exclusion of such a small number of individuals would have altered our results. We used the Gower’s dissimilarity matrix given the categorical nature of data, as this is appropriate for such data.*

(e) Statistical analysis: "Multivariable" logistic regression was used. The correct word is "Multiple" logistic regression, because multivariable would mean something different. This change needs to be made throughout the manuscript. Furthermore, some goodness-of-fit assessments after performing the multiple logistic regressions (say, via the Hosmer-Lemeshow statistics) is desirable.

*Thank you, we have updated this as suggested but multivariable and multiple logistic regression are different words for the same (*[*https://www.ncbi.nlm.nih.gov/pmc/articles/PMC3518362/*](https://www.ncbi.nlm.nih.gov/pmc/articles/PMC3518362/)*) (line 212 in the clean and 226 in the tracked version).*
