# Supplementary material for: Characteristics and impact of Long Covid: Findings from an online survey
Source: PLoS One. 2022 Mar 8;17(3):e0264331. doi: 10.1371/journal.pone.0264331 (PMC8903286; doi:10.1371/journal.pone.0264331)
Supplement: Attachment — Submitted filename: Response_to_reviewers_R2.docx [file pone.0264331.s015.docx]

ACADEMIC EDITOR: The authors have adequately addressed the reviewer's comments. Before the paper can be accepted, please address the following minor issues:

1. Abstract - Methods: Suggest revising to “We collected self-reported data through an online survey using convenience non-probability sampling. The survey enrolled adults with lab-confirmed (PCR or antibody) or suspected COVID-19 who were not hospitalized in the first two weeks of illness. This analysis was restricted to those with self-reported Long Covid. Univariate comparisons..."

*Thank you, we have revised as suggested.*

2. Introduction: typo on line 108 of tracked version - “perceived a lack of data on COVID-19 sequelae…”

*Thank you, we have corrected this typo.*

3. Introduction – last paragraph: suggest revising line 110 in tracked version to "In adults who self-reported Long Covid after suspected or confirmed COVID-19 and..."

*Thank you, we have revised as suggested.*

4. Methods – last paragraph: suggest revising line 238 in tracked version to “As the full analysis included those with and without lab-confirmed diagnosis of COVID-19, we examined whether this was a significant predictor of cluster membership to assess whether clusters correlated with having lab-confirmation of infection. We also carried out an additional sensitivity analysis by clustering only those with lab confirmation to see if clusters obtained were different from the full sample analysis.”

*Thank you, we have revised as suggested.*

5. Results, new sentence in line 256-258 of tracked version: Based on Table 1, I think you mean: “The proportion of participants from outside the UK was higher among those with lab-confirmed infection (29.1%) than among those with suspected infection (17.0%).”

*Thank you, we have revised as suggested.*

6. Results – first paragraph: can the authors add in the median time from symptom onset to completing the survey (presumably this is different from reported duration of illness since some people have recovered). I am also assuming that duration of illness was just the time from symptom onset to completing the questionnaire for those still symptomatic at survey completion (if this is incorrect, then please clarify in the methods how this was calculated)- was the longer duration of illness among negatives simply due to a longer time since survey completion?

*Thank you, we have updated the median and IQR to present these after excluding those who have recovered (in the abstract and the results section). The mean and SD are unchanged. We have also updated the n in Table 4 where we have presented mean duration of illness.*

*Yes, the duration of illness was calculated as the time from symptom onset to the date of survey completion for those still symptomatic.*

*The longer duration among negatives/not tested is likely to reflect earlier symptom onset when access to testing (in the UK) was limited.*

7. Table 1 and 2 footnote: revise to “Comparisons between those with and without lab-confirmed COVID-19 used...”

*Thank you, we have revised the footnote for Tables 1-6 as suggested.*

8. Results – previous health: revise last sentence to “There were no significant differences in these proportions between those with and without lab-confirmed infection”.

*Thank you, we have revised as suggested.*

9. Results, course of illness: revise line 287-288 of tracked version to “…which were higher in those with lab confirmation than without, whereas abdominal pain, nausea, chest pain, chest tightness, chills, hoarse voice, sore throat, sneezing and pins and needles were lower in those with lab-confirmation than without.”

*Thank you, we have revised as suggested.*

10. Table 4: ‘Comes and goes’ and ‘relapsing’ are listed separately in the table but reported/defined together in the text (line 314). How are these different?

*Thank you, we have combined these into one category.*

*‘Comes and goes’ was one of the options included in our survey but we also gave participants an ‘other’ option which they could choose and describe the pattern of their illness if they didn’t feel the available options captured the pattern they experienced. The responses to the other option were then categorised by the research and coded as a separately relapsing category.*

11. Results – lab confirmation of infection: Move sentence (starting with “1172 participants (46%) reported having an antibody test…”) on line 381 of tracked version to the beginning of that paragraph on antibody testing. Also add a period between ‘test’ and ’26.3%’ on line 379.

*Thank you, we have revised as suggested.*

12. Table S4 – many of the categories are overlapping – please revise.

*Thank you, we have updated this to clarify the categories.*

13. Results – lab confirmation of infection: the last paragraph seems redundant with the prior sections since differences by lab confirmation are now presented throughout – suggest deleting it.

*Thank you, we have moved two of the sentences that were not previously covered in the text and deleted the rest of the paragraph as suggested.*

14. Results, clustering: second paragraph describing results of univariate analysis – for some the % are presented in the text (e.g. for loss of income)- suggest removing to be consistent for all results mentioned.

*Thank you, we have revised as suggested.*

15. Results, clustering: last paragraph, correct reference to “OR” for ‘having a confirmed positive test’.

*Thank you, we have corrected this as suggested.*
